# SUMO-targeted ubiquitin ligase activity can either suppress or promote genome instability, depending on the nature of the DNA lesion

**DOI:** 10.1371/journal.pgen.1006776

**Published:** 2017-05-05

**Authors:** Minghua Nie, Bettina A. Moser, Toru M. Nakamura, Michael N. Boddy

**Affiliations:** 1 Department of Molecular Medicine, The Scripps Research Institute, La Jolla, CA, United States of America; 2 Department of Biochemistry and Molecular Genetics, College of Medicine, University of Illinois at Chicago, Chicago, IL, United States of America; Duke University, UNITED STATES

## Abstract

The posttranslational modifiers SUMO and ubiquitin critically regulate the DNA damage response (DDR). Important crosstalk between these modifiers at DNA lesions is mediated by the SUMO-targeted ubiquitin ligase (STUbL), which ubiquitinates SUMO chains to generate SUMO-ubiquitin hybrids. These SUMO-ubiquitin hybrids attract DDR proteins able to bind both modifiers, and/or are degraded at the proteasome. Despite these insights, specific roles for SUMO chains and STUbL in the DDR remain poorly defined. Notably, fission yeast defective in SUMO chain formation exhibit near wild-type resistance to genotoxins and moreover, have a greatly reduced dependency on STUbL activity for DNA repair. Based on these and other data, we propose that a critical role of STUbL is to antagonize DDR-inhibitory SUMO chain formation at DNA lesions. In this regard, we identify a SUMO-binding Swi2/Snf2 translocase called Rrp2 (ScUls1) as a mediator of the DDR defects in STUbL mutant cells. Therefore, in support of our proposal, SUMO chains attract activities that can antagonize STUbL and other DNA repair factors. Finally, we find that Taz1^TRF1/TRF2^-deficiency triggers extensive telomeric poly-SUMOylation. In this setting STUbL, together with its cofactor Cdc48^p97^_,_ actually promotes genomic instability caused by the aberrant processing of *taz1Δ* telomeres by DNA repair factors. In summary, depending on the nature of the initiating DNA lesion, STUbL activity can either be beneficial or harmful.

## Author summary

Since its discovery in 2007, SUMO-targeted ubiquitin ligase (STUbL) activity has been identified as a key regulator of diverse cellular processes such as DNA repair, mitosis and DNA replication. In each of these processes, STUbL has been shown to promote the chromatin extraction and/or degradation of SUMO chain modified proteins. However, it remains unclear whether STUbL acts as part of a "programmed" cascade to remove specific proteins, or antagonizes localized SUMO chain formation that otherwise impedes each process. Here we determine that SUMO chains, the major recruitment signal for STUbL, are largely dispensable for genotoxin resistance in fission yeast. Moreover, when SUMO chain formation is compromised, the need for STUbL activity in DNA repair is strongly reduced. These results indicate a primary role for STUbL in antagonizing localized SUMO chain formation. Interestingly, we also find that STUbL activity can be toxic at certain genomic lesions that induce extensive local SUMOylation. For example, STUbL promotes the chromosome instability and cell death caused by deprotected telomeres following Taz1^TRF1/2^ deletion. Together, our data suggest that STUbL limits DNA repair-inhibitory SUMO chain formation, and depending on the nature of the genomic lesion, can either suppress or cause genome instability.

## Introduction

The Small Ubiquitin-like Modifier (SUMO) is a posttranslational modifier (PTM) that critically regulates most aspects of cell growth. For example, key roles for SUMOylation in genome stability, transcription, proteostasis, and embryonic development have been identified [1–11].

In fission yeast, SUMO is covalently attached to lysine residues within its targets via a conserved enzymatic cascade of E1 activating (Fub2:Rad31 heterodimer), E2 conjugating (Ubc9), and E3 ligase factors (Pli1, Nse2; [1]). Pli1 and Nse2 drive the majority of SUMOylation, and are members of the evolutionarily conserved PIAS class of SUMO E3 ligases [12–14].

Pli1-dependent SUMOylation requires a non-covalent SUMO:Ubc9 complex [12], which is also required to generate SUMO chains by the sequential addition of SUMO to N-terminal lysine residues of SUMO (K14 and K30 in fission yeast; [12, 15–17]). Interestingly, despite catalyzing more than 90% of cellular SUMOylation, deletion of Pli1 in fission yeast does not cause overt growth defects or sensitivity to genotoxins [12, 18]. However, cells lacking Pli1 have defects in centromere silencing, and also have elongated telomeres due to the loss of Tpz1 SUMOylation [14, 18–20]. Despite a relatively restricted target pool, Nse2-mediated SUMOylation is required for genotoxin resistance and genome stability, but not telomere length control [12–14, 21–24].

Countering the activities of Pli1 and Nse2, the SUMO proteases Ulp1 and Ulp2 (also called SENPs) remove SUMO from target proteins, and disassemble poly-SUMO chains [25–27]. Deletion of Ulp2 causes a striking accumulation of SUMO conjugates, especially high molecular weight (HMW) species that correspond to SUMO chains [28]. Abrogating SUMO chain formation by mutating SUMO's N-terminal acceptor lysine residues rescues many of the severe phenotypes caused by Ulp2 deletion, which include sensitivity to various genotoxic agents, genome instability and temperature sensitivity [12, 28]. Moreover, deletion of Pli1, which catalyzes SUMO chain formation, also rescues *ulp2Δ* phenotypes [29]. Importantly therefore, in contrast to global hypoSUMOylation e.g. in *pli1Δ* cells, uncontrolled SUMO chain formation has relatively dire consequences for the cell.

SUMO chains are also key substrates for the SUMO-targeted ubiquitin ligase (STUbL) family of E3 ubiquitin ligases [30–34]. STUbLs ubiquitinate SUMO chains, both “capping” them [35] and creating a dual signal for the recruitment of proteins able to simultaneously bind both SUMO and ubiquitin e.g. the AAA ATPase p97/Cdc48-Ufd1-Npl4 that remodels chromatin-associated complexes, and the BRCA1 cofactor RAP80 [36, 37].

STUbL and p97/Cdc48 together constitute a mechanism for the extraction and/or degradation of SUMO/ubiquitin modified proteins from chromatin or other protein complexes [36, 38–40]. For example, the SUMOylated and chromatin bound fraction of FANCI, a Fanconi Anemia complex protein, is controlled by STUbL and p97-dependent extraction from chromatin and proteasomal degradation [41]. In addition, the poly-SUMOylated forms of the DDR factors MDC1 and RPA are also subject to STUbL-dependent regulation at DNA lesions [42–45]. The accumulation of poly-SUMOylated MDC1 and RPA at lesions in STUbL deficient cells correlates with defects in downstream events in the DDR e.g. Rad51 loading, and a blockade to homologous and non-homologous recombination repair. In these studies SUMO conjugation defective mutants of each factor were used to infer the impact of their poly-SUMOylation and STUbL-dependent processing on DNA repair. However, such mutants are refractory to mono- and poly-SUMOylation, as well as other possible lysine modifiers including ubiquitin. Therefore, questions remain about the specific contribution(s) of SUMO chains and STUbL in regulating the DDR.

As mentioned, Pli1-dependent SUMOylation of Tpz1 regulates telomere length homeostasis [19, 20]. SUMOylation also controls the cold sensitive phenotype of fission yeast lacking the telomere shelterin factor Taz1 (human TRF1/2) [46]. Specifically, *taz1Δ* cells are cold sensitive due to telomere "entanglement" and subsequent chromosome breakage during mitosis; phenotypes that can be prevented by reducing SUMOylation of the RecQ DNA repair helicase Rqh1 [46, 47]. Thus, SUMOylated Rqh1 is toxic to cells lacking Taz1, but potential roles for SUMO chains and STUbL in this toxic DNA repair process remain untested.

Overall, diverse STUbL targets have been identified, and the SUMO-dependent degradation of proteins appears to be pivotal for the output of a number of cellular pathways, including DNA repair [7, 38, 48]. However, if the SUMO-dependent degradation of certain DNA repair proteins was required, then preventing SUMO chain formation should yield phenotypes similar to those of cells lacking STUbL i.e. genome instability and lethality [30–34, 42, 44, 45, 49]. On the contrary, cells containing SUMO chain blocking mutations lack overt phenotypes in fission yeast e.g. SUMO^K14,30R^ [12], and analogous SUMO mutants in budding yeast have very mild phenotypes when compared to STUbL deficiency [28, 50].

In light of the above data, we hypothesized that a critical physiological role of STUbL in the DNA damage response (DDR) is to prevent a local toxic buildup of SUMO chains. By manipulating SUMO pathway homeostasis and assessing the DNA damage response (DDR) to distinct genomic lesions we obtain key support for the above model. Furthermore, we identify the SUMO-interacting Swi2/Snf2 family DNA translocase, Rrp2, a candidate mediator of SUMO chain toxicity in STUbL mutant cells. Finally, in addition to the known positive functions of STUbL, we show that at certain DNA lesions its activity can actually cause genome instability.

## Results

### STUbL antagonizes the nucleation of SUMO chains

**Fig 1:**
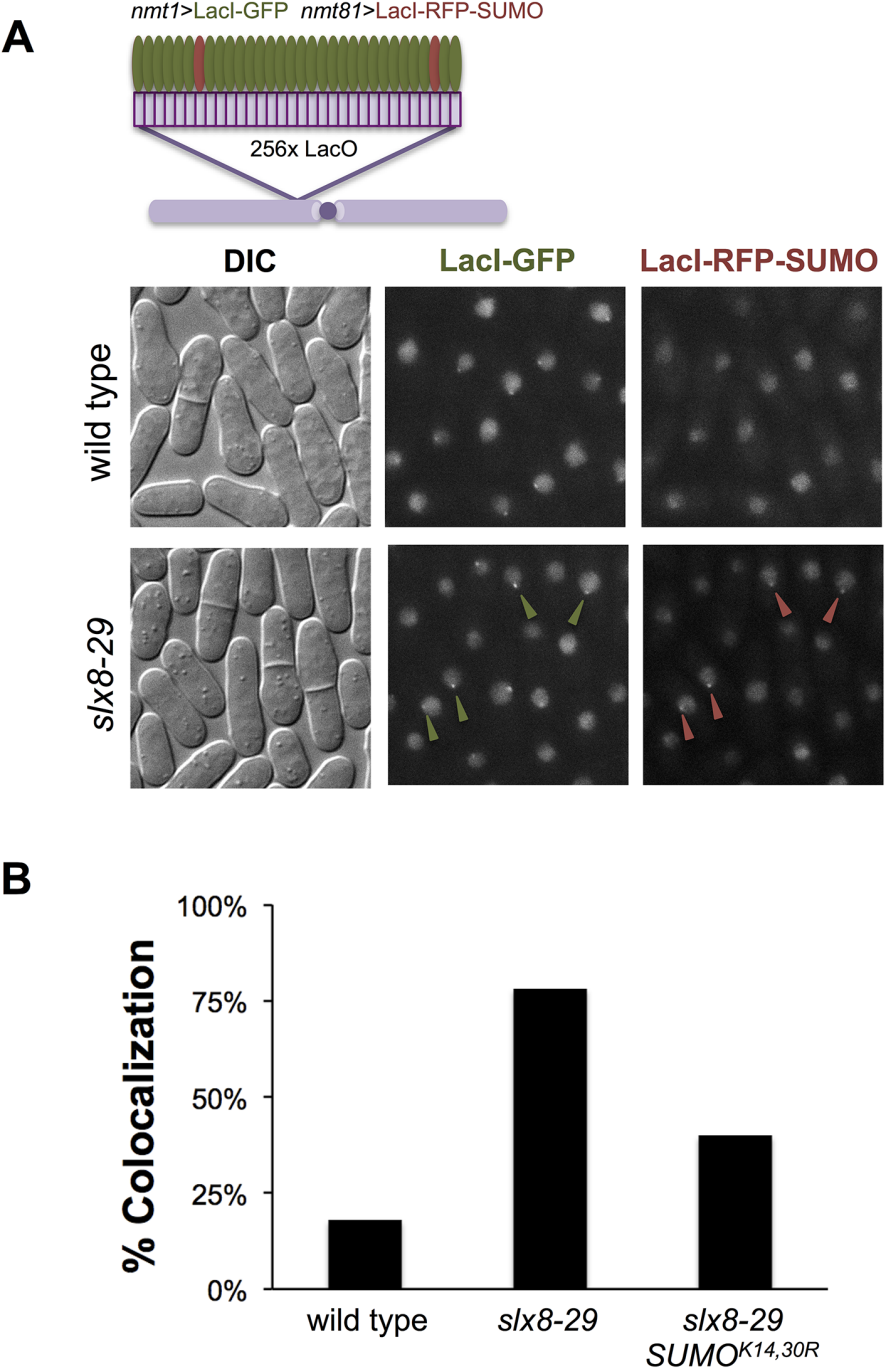
Forced nucleation of SUMO on chromatin engages STUbL activity. *A*, Wild-type or *slx8-29* cells with 256-repeats of the LacO sequence integrated near *cen1* that express LacI-GFP from the P_*nmt1*_ promoter and LacI-mCherry-SUMO from the attenuated P_*nmt81*_ promoter. Cells were grown overnight at 30°C in filtered EMM-LUAH with 5x Thiamine (B1) to allow leaky protein expression from the *nmt* promoters, and imaged live. *B*, Percentage of cells containing co-localized mCherry and GFP foci over total cells containing GFP foci was plotted against each genotype. Over 200 cells were counted for each genotype.

We previously showed that SUMO chains spontaneously accumulate in a single large subnuclear focus in STUbL mutant cells, suggesting that STUbL prevents SUMO pathway nucleation and rampant SUMO chain formation [36]. To test this more directly, we expressed both LacI-RFP-SUMO and LacI-GFP in fission yeast that have a cluster of LacO repeats integrated at the *lys1* locus of chromosome 1 (~256 repeats, [51]). LacI-GFP was expressed from the *nmt1* promoter whereas LacI-RFP-SUMO was expressed from the attenuated *nmt81* promoter, both under repressive conditions (+ thiamine [52]). This sets up a competition wherein the more abundant LacI-GFP protein occupies the LacO repeats, forming visible foci and largely excluding LacI-RFP-SUMO (Fig 1A).

In wild-type cells, as anticipated the LacI-GFP protein can be readily visualized as a focus on the LacO repeats, whereas LacI-RFP-SUMO is diffuse and pan-nuclear (Fig 1A). In contrast, LacI-GFP and LacI-RFP-SUMO are both frequently present in co-localizing nuclear foci in *slx8-29* cells that have compromised STUbL activity (Fig 1A). Quantification revealed that LacI-GFP and LacI-RFP-SUMO foci colocalize in ~18% of wild-type cells, and this colocalization was strikingly increased to ~80% in *slx8-29* cells, consistent with a role for STUbL in antagonizing SUMO chain nucleation (Fig 1B). Notably, expression of LacI-RFP-SUMO^K14,30R^ with LacI-GFP reduced the colocalization of GFP and RFP signals to ~40% in *slx8-29* cells, indicating that the observed SUMO foci contain SUMO chains. That LacI-RFP-SUMO^K14,30R^ expression does not completely suppress focus formation to the levels of LacI-RFP-SUMO foci in wild-type cells is expected based on the residual limited capacity of SUMO^K14,30R^ to form SUMO chains through alternative internal lysine residues [36]. Therefore, STUbL can antagonize SUMO chains when they are nucleated on chromatin, even in the absence of active DDR signaling.

### "Low dose" SUMO suppresses STUbL mutant genotoxin sensitivity

**Fig 2:**
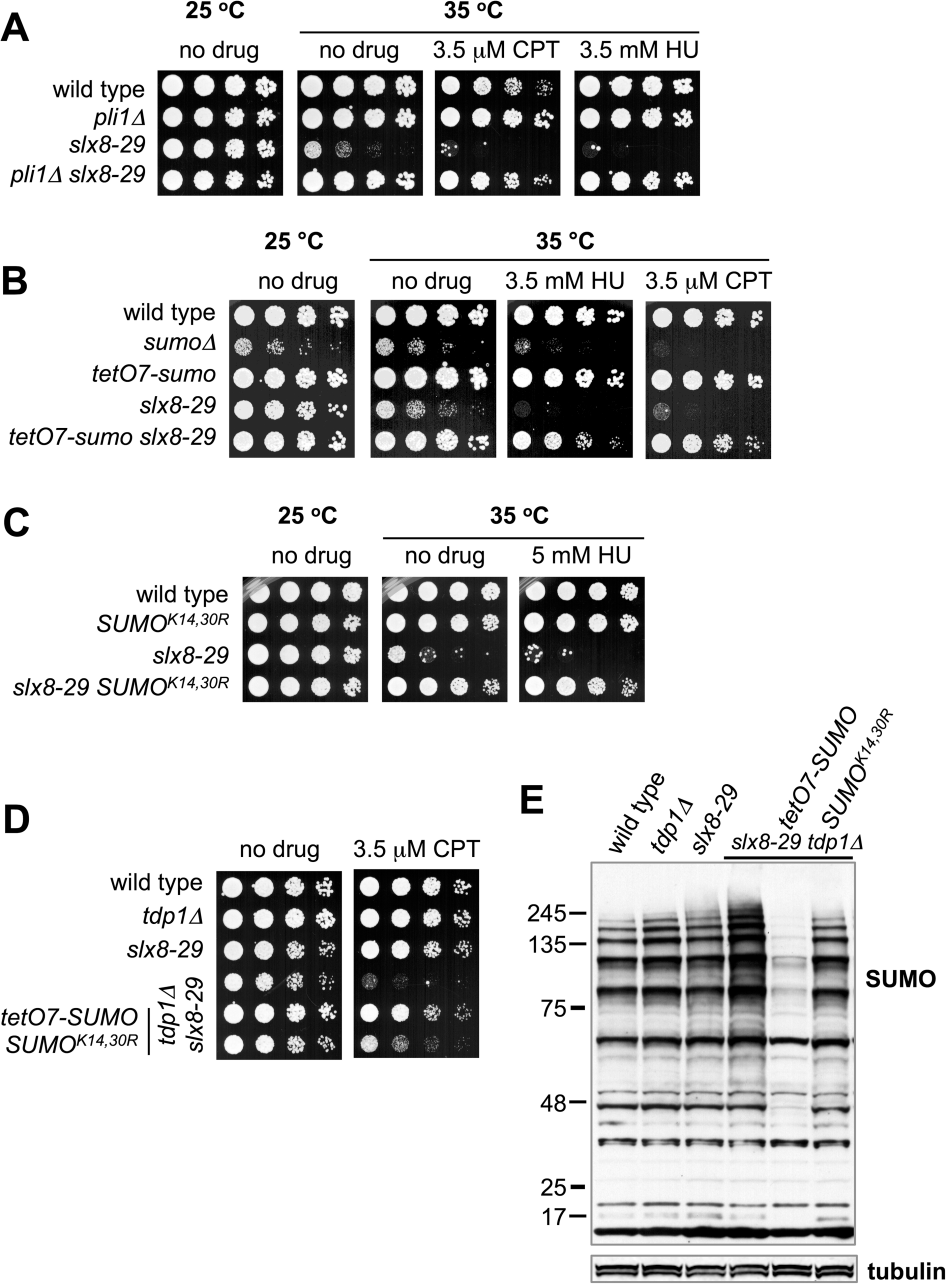
STUbL mutant phenotypes are mitigated by mutations that counteract SUMO chain accumulation. *A* to *D*, five fold serial dilutions of the indicated strains were spotted onto YES plates, with or without the indicated genotoxic challenge, and grown at indicated temperature. Cells in *D* were grown at 25°C. *E*, anti-SUMO and tubulin Western blots of total proteins from the indicated strains grown at 25°C to log phase.

If countering SUMO chain nucleation and growth is a key function of STUbL, then reducing SUMO chain formation should lessen cellular dependency on STUbL. Indeed, deleting the SUMO chain catalyst Pli1 strongly suppresses STUbL mutant phenotypes, and also makes the normally essential fission yeast STUbL dispensable for growth (Fig 2A & [30]).

We next asked if simply reducing the levels of wild-type SUMO would make it limiting for chain formation, thereby phenocopying *pli1Δ*. To this end, we replaced the endogenous SUMO promoter with a weaker constitutive promoter [53]. In stark contrast to *sumoΔ* cells, those expressing less SUMO (*tetO7-SUMO*) appear wild-type for growth and genotoxin resistance (Fig 2B).

We then determined the effect of *tetO7-SUMO* on cells with compromised STUbL activity. Strikingly, *tetO7-SUMO* potently rescued the temperature and genotoxin sensitivity of *slx8-29* cells (Fig 2B). Under conditions where *slx8-29* cells are completely growth inhibited, *tetO7-SUMO slx8-29* double mutants grow almost as well as *tetO7-SUMO* single mutants. In addition, *tetO7-SUMO* similarly suppressed the genotoxin sensitivity of *ulp2Δ* cells, which lack the SUMO chain editing protease Ulp2 (S1A Fig, [28]). The phenotypic rescues of *slx8-*29 *and ulp2Δ* cells were accompanied by a reduction in the HMW SUMO chains that normally accumulate in these mutants (S1B Fig, [54]). Supporting the SUMO chain specificity of *slx8-29* suppression by *pli1Δ* and *tetO7-SUMO*, the SUMO^K14,30R^ chain mutant also suppresses *slx8-29* (Fig 2C, [12]).

We previously identified a role for STUbL in the repair of topoisomerase 1 (Top1)-dependent lesions [22]. In the absence of Tdp1, an enzyme that catalyzes the reversal of Top1-DNA adducts, cells require STUbL activity for removal of Top1-DNA adducts via a parallel repair pathway (Fig 2D, [22]). This dependency can be partially alleviated by introducing the SUMO chain reducing mutant SUMO^K14,30R^ (Fig 2D). Notably, *tetO7-SUMO* is more effective than SUMO^K14,30R^ at rescuing the synthetic *slx8-29 tdp1Δ* phenotype (Fig 2D). This difference may be due to the residual SUMO chain-forming capacity of SUMO^K14,30R^ [12, 36]. HMW SUMO species in *slx8-29 tdp1Δ* cells are reduced by *tetO7-SUMO* and SUMO^K14,30R^, correlating with the extent of rescue (Fig 2E).

Overall, these data indicate that "low dose SUMO" (*tetO7-SUMO*) limits toxic Pli1-dependent SUMO chain formation in STUbL mutant cells, similar to but more potently than SUMO^K14,30R^. It is somewhat surprising that SUMO chain formation follows an apparently passive mechanism, likely driven by the normally high local concentrations of SUMO. In this setting, local pathway control by STUbL and deSUMOylating enzymes would appear critical. Such a role for STUbL is well supported by the focal accumulation of SUMO chains in fission yeast STUbL mutants (Fig 1 & [36]).

### SUMO-binding Swi2/Snf2 translocase Rrp2 is toxic to STUbL mutant cells

Our data are consistent with SUMO chains in STUbL mutant cells exerting an inhibitory effect on DNA repair. To identify potential SUMO chain "effectors", we purified fission yeast proteins that have affinity for SUMO chains [36]. In this way we made the initial identification of Cdc48-Ufd1-Npl4 as a STUbL co-factor that recognizes both SUMO and ubiquitin modifications on its targets [36].

Another SUMO chain interactor we identified was the Swi2/Snf2-related DNA translocase called Rrp2 in fission yeast and Uls1 in budding yeast. Both Rrp2 and Uls1 bind SUMO [32, 55], and Uls1 has been described as a STUbL due to its interaction with SUMO chains and the presence of an E3 ubiquitin ligase RING domain (although ubiquitin ligase activity is yet to be demonstrated) [32, 56]. An elegant study in budding yeast suggested that Uls1 could be an antagonist of the Slx8-based STUbL [56], but straightforward interpretation of this model was complicated by the synthetic sickness of *slx8Δ* and *uls1Δ* [32, 57, 58].

**Fig 3:**
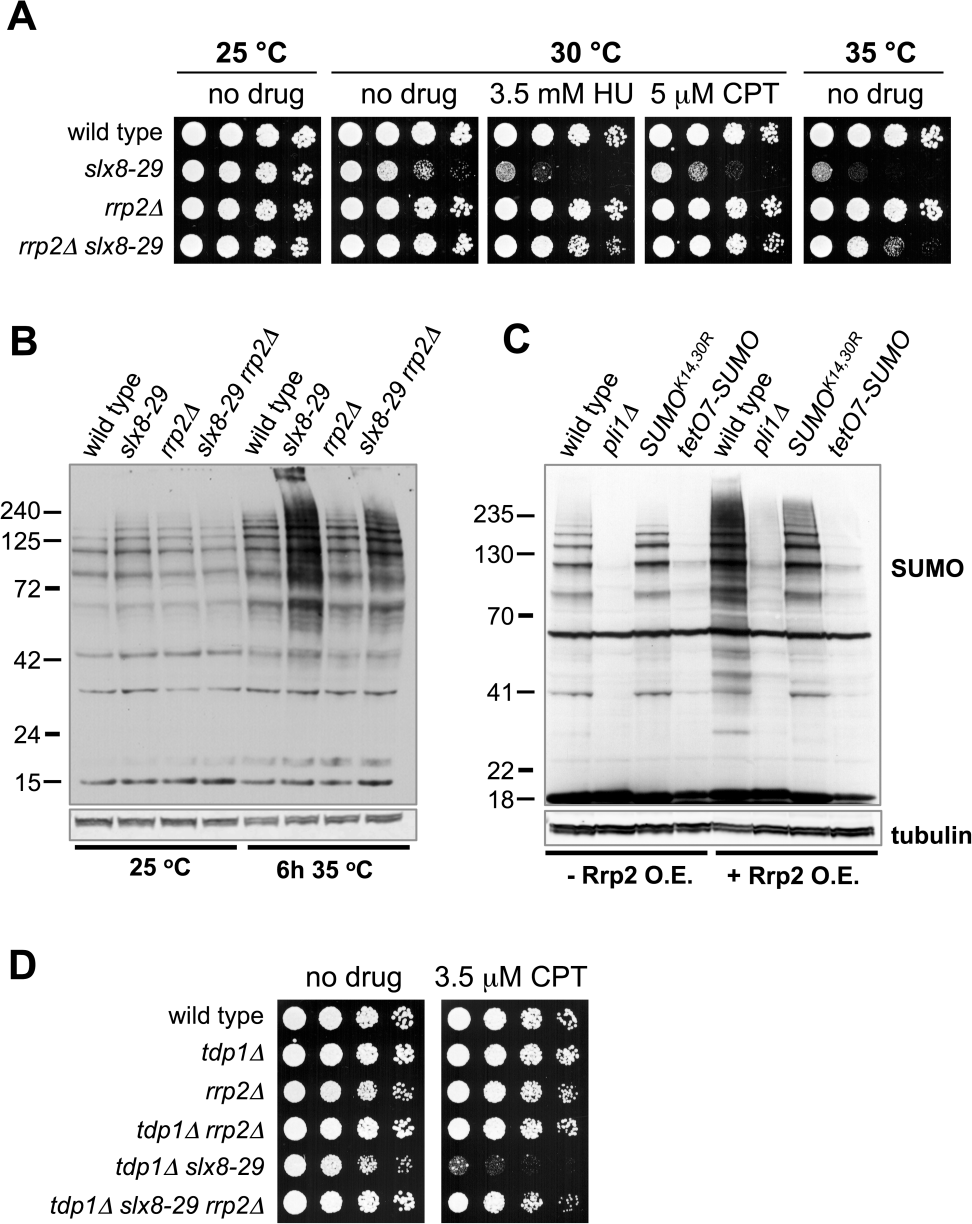
STUbL mutant phenotypes are mitigated by Rrp2 deletion. *A*, five fold serial dilutions of the indicated strains were spotted onto YES plates, with or without hydroxyurea (HU) or camptothecin (CPT), and grown at the indicated temperatures. *B* & C, anti-SUMO and tubulin Western blots of total proteins of indicated strains grown in YES (*B*) or minimal selection (UAH) medium (*C*). To induce Rrp2 overexpression (Rrp2 O.E.), cells containing pREP41-mCherry-FLAG-Rrp2 were grown in the absence of Thiamine (B1) for 24 h. *D*, five fold serial dilutions of the indicated strains were spotted onto YES plates, with or without camptothecin (CPT), and grown at 25°C.

In light of the foregoing, we tested for a genetic interaction between the *slx8-29* and *rrp2Δ* mutations. Deletion of Rrp2 did not cause overt phenotypes such as temperature or genotoxin sensitivity (Fig 3A). Strikingly however, *rrp2Δ* instead suppressed the temperature and genotoxin sensitivities of *slx8-29* cells (Fig 3A). Suppression of *slx8-29* phenotypes by *rrp2Δ* was accompanied by a reduction in the levels of HMW SUMO conjugates (Fig 3B). On the other hand, ectopic expression of Rrp2 strongly induced the formation of HMW SUMO species, in wild-type but not SUMO chain deficient backgrounds (Fig 3C). Moreover, deletion of Rrp2 mitigated the synthetic sickness and camptothecin sensitivity of *slx8-29* and *tdp1Δ* mutations (Fig 3D).

The simplest interpretation of these data is that Rrp2 inhibits SUMO chain-directed STUbL activity, which is further supported by the analysis of crosstalk between Uls1 and STUbL in budding yeast [56]. Together with the known functions for Rrp2 and Uls1 in modulating DNA repair [55, 59–61], our results reveal Rrp2 as an excellent candidate mediator of the DDR-inhibitory effects of SUMO chains in *slx8-29* cells.

### Impact of SUMO chains on the viability of cells lacking Taz1

The telomeres of cells lacking Taz1 are elongated and become "entangled" at low growth temperatures causing chromosome breakage during mitosis, G2 cell cycle checkpoint activation, and cell death [46, 47, 62]. The precise mechanism of *taz1Δ* telomere entanglement is unknown. However, it is promoted by DNA processing factors such as the RecQ DNA helicase Rqh1 and the DNA exonuclease Exo1 [46, 63], and can be suppressed by certain Topoisomerase II mutations [64]. Interestingly, all of these factors are regulated by SUMO conjugation [46, 65–67], and reducing Rqh1 SUMOylation suppresses *taz1Δ* telomere entanglement [46]. Notably, the impact of SUMO chains on *taz1Δ* phenotypes has not been determined. Therefore, we assessed the effects of a panel of SUMO pathway-related mutants on the cold sensitivity of *taz1Δ* cells.

**Fig 4:**
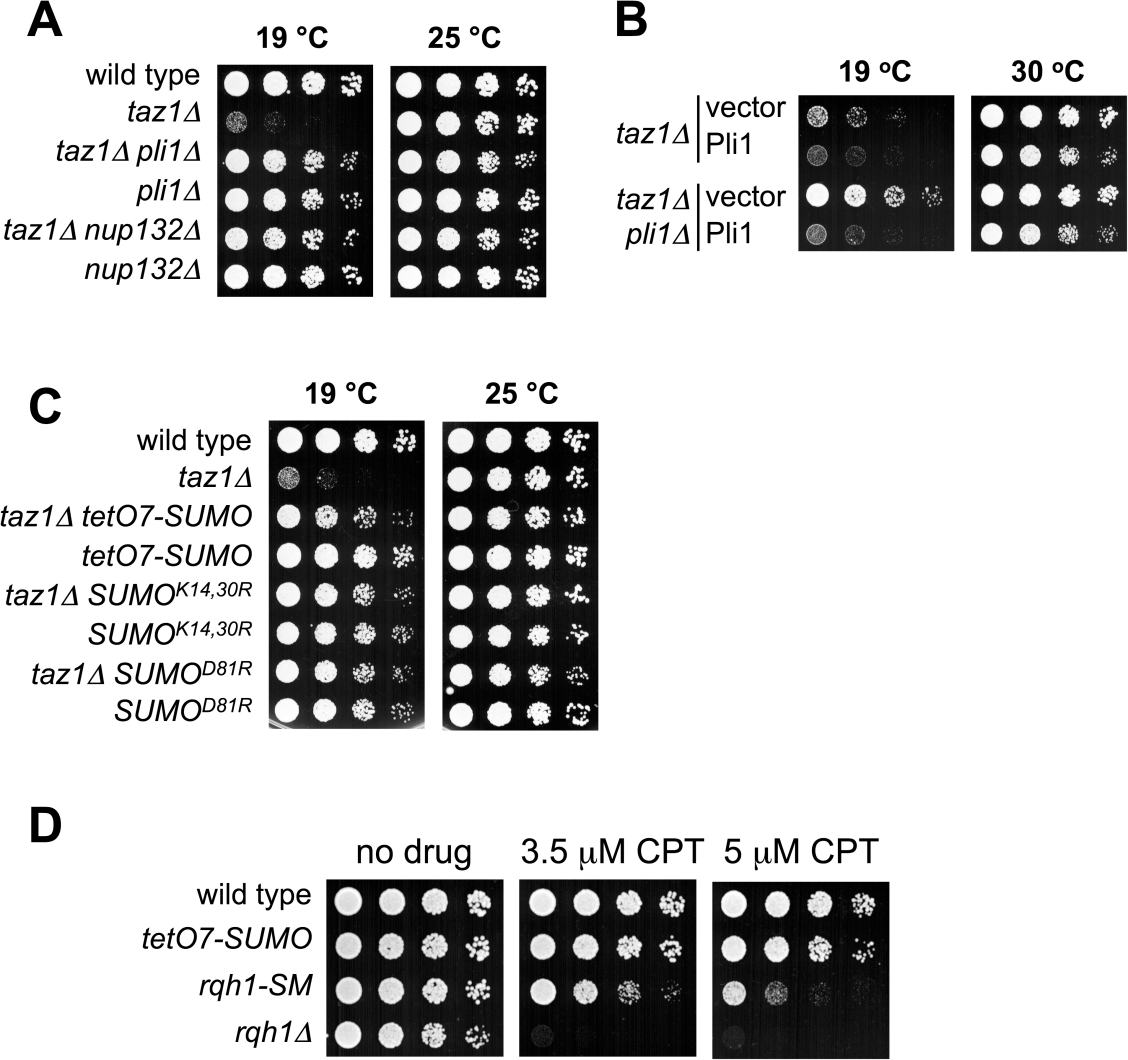
Rescue of *taz1Δ* cold sensitivity by mutations that reduce SUMO chain formation. *A* to *D*, five fold serial dilutions of the indicated strains were spotted onto YES plates, the cells were incubated at 25 or 19°C for three to six days. *B*, dilution series of the indicated strains were spotted onto minimum medium (EMM-UAH) without thiamine (B1) to induce the expression of Pli1 and incubated at 19°C or 30°C. Cells in *D* were grown at 30°C.

Strikingly, *pli1Δ taz1Δ* and *nup132Δ taz1Δ* double mutant cells grow well at low temperature compared to *taz1Δ* cells (Fig 4A). Previously, Pli1 deletion was suggested to not suppress *taz1Δ* cold sensitivity [46]. Therefore, we further validated our results by re-expressing Pli1 in *pli1Δ taz1Δ* cells, which confirmed that Pli1 activity promotes *taz1Δ* cold sensitivity (Fig 4B). The similar effect of deleting Pli1 or Nup132 on *taz1Δ* cold sensitivity likely reflects the STUbL-mediated degradation of Pli1 in *nup132Δ* cells [29]. Finally, SUMO^D81R^ that abolishes the SUMO:Ubc9 non-covalent complex and reduces Pli1-dependent SUMOylation also suppresses *taz1Δ* (Fig 4C, [12]).

We previously showed that Pli1 has separable roles in mono-SUMOylation and SUMO chain formation [12]. We therefore asked which function of Pli1 causes *taz1Δ* cold sensitivity. Notably, reducing SUMO chain formation with either the SUMO^K14,30R^ or *tetO7-SUMO* allele also rescues *taz1Δ* cold sensitivity to a similar degree as *pli1Δ* (Fig 4C). Therefore, Pli1-dependent SUMO chains, not mono-SUMOylation events, cause *taz1Δ* cold sensitivity.

Reducing Rqh1 SUMOylation has been shown to rescue *taz1Δ* cold sensitivity [46]. Therefore, we considered that reduced Rqh1 SUMOylation could explain the rescue of taz1*Δ* phenotypes by tetO7-SUMO. Notably however, the Rqh1-SM SUMOylation defective mutant renders cells camptothecin sensitive [46], whereas *tetO7-SUMO* cells are not (Fig 4D). Therefore, Rqh1 SUMOylation is intact in *tetO7-SUMO* cells, as are other key SUMOylation events that control genotoxin resistance (e.g. Fig 2B & 2D), and telomere length e.g. Tpz1 SUMOylation (see below).

### STUbL and Cdc48/p97 promote genome instability in *taz1Δ* cells

**Fig 5:**
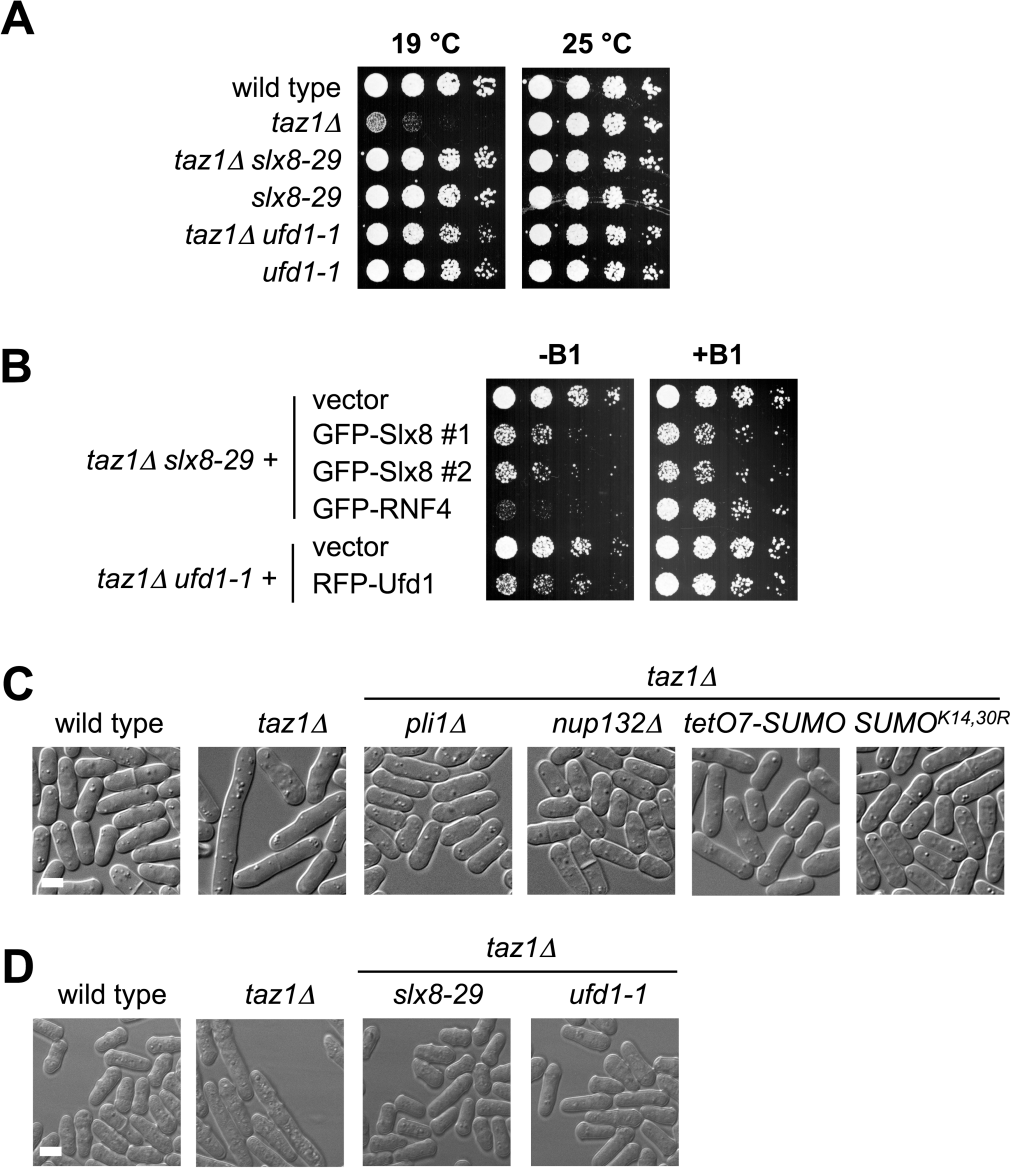
Rescue of *taz1Δ* cold sensitivity and checkpoint activation by STUbL and Cdc48/p97 mutants. *A*, five fold serial dilutions of the indicated strains were spotted onto YES plates, and incubated at 25 or 19°C for three to six days. *B*, dilution series of the indicated strains were spotted onto minimum medium (EMM-UAH) with or without thiamine (B1) and incubated at 19°C for six days. *C* & *D*, differential interference contrast (DIC) light microscopy of the indicated strains grown for two days in liquid culture at 19°C. *Bar*, 5 μM.

Based on the suppression of *taz1Δ* phenotypes by SUMO pathway mutants that compromise SUMO chain formation, we tested if STUbL activity plays a role in this context. As can be seen, *slx8-29 taz1Δ* double mutant cells grow robustly at low temperature, unlike the *taz1Δ* single mutant (Fig 5A). Moreover, *ufd1-1*, a mutation that compromises the Cdc48/p97-dependent processing of STUbL targets [36] also rescues *taz1Δ* cold sensitivity (Fig 5A). Expression of either Slx8 or the human STUbL RNF4 reverses the suppression of *taz1Δ* by *slx8-29*, as does expression of Ufd1 in *ufd1-1 taz1Δ* cells (Fig 5B). Therefore, SUMO chain-dependent STUbL and Cdc48(p97) activity has pathological consequences at *taz1Δ* telomeres.

At low temperatures *taz1Δ* cells undergo checkpoint-dependent cell cycle arrest due to chromosome breakage in mitosis [47]. In fission yeast, cell cycle arrest is readily visualized as an increase in cell length. Therefore, we compared cell lengths of wild-type, *taz1Δ* and double mutants including *tetO7-SUMO taz1Δ*, *slx8-29 taz1Δ* and *ufd1-1 taz1Δ* at 19°C. As can be seen, *taz1Δ* cells are highly elongated compared to each of the double mutants, which are instead similar to wild-type in length (Fig 5C & 5D). Given that these mutants are checkpoint proficient, SUMO chains, STUbL and Cdc48/p97 activity drive the chromosome damage and checkpoint activation in *taz1Δ* cells [22, 36].

### Distinct effects of SUMO pathway mutants on telomere length

**Fig 6:**
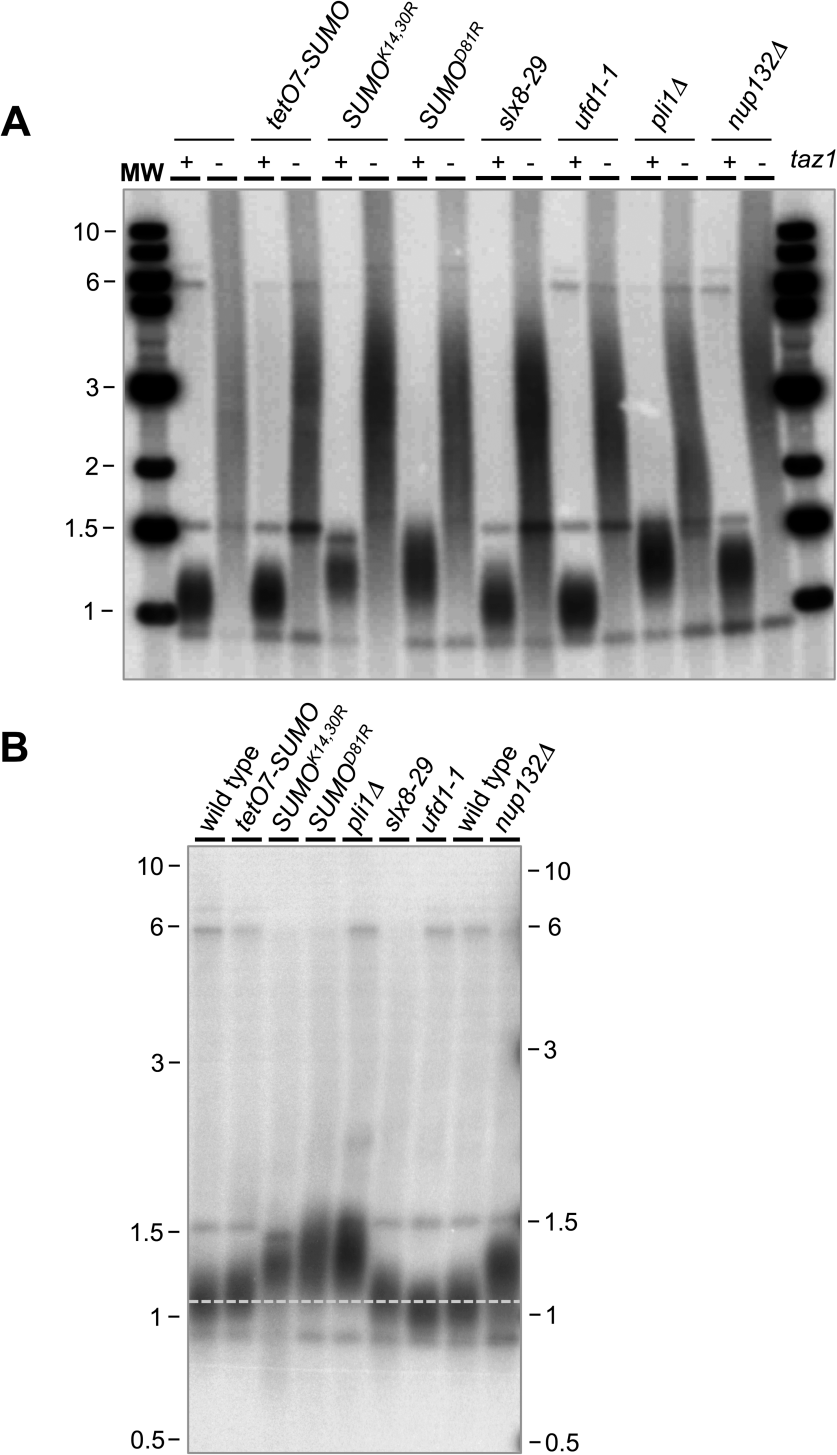
The effect of SUMO pathway mutations on telomere length. *A*, genomic DNA samples of the indicated genotypes grown for two days at 19°C were analyzed for telomere length by Southern blot hybridization with a telomere DNA probe. *B*, DNA samples of the indicated genotypes grown at 25°C were analyzed for telomere length by Southern blot hybridization with a telomere DNA probe.

As the SUMO pathway controls telomere length [14, 18–20], we asked if suppression of *taz1Δ* phenotypes is due to restoration of normal telomere homeostasis in the SUMO pathway and *taz1Δ* double mutants e.g. *slx8-29 taz1Δ*. However, telomere length analysis revealed no overt effects on telomere length, with telomeres in each double mutant being highly elongated, as in *taz1Δ* cells (Fig 6A). This finding is similar to that reported for the Rqh1 SUMOylation mutant Rqh1-SM, which also rescues *taz1Δ* cold sensitivity without affecting *taz1Δ* telomere length [46].

Recently, Pli1-dependent SUMOylation of the telomere factor Tpz1 was shown to antagonize telomerase activity by recruiting the Stn1-Ten1 complex to telomeres [19, 20]. Therefore, deletion of Pli1 causes telomere elongation, which can be readily visualized by Southern analysis of chromosome termini (Fig 6A, [14, 18–20]). Interestingly, in addition to the expected telomere elongation in *pli1Δ* cells, our Southern analyses indicated telomere homeostasis defects in some other SUMO pathway mutants (Fig 6A), which we analyzed further.

We have shown that SUMO^D81R^ abolishes the non-covalent SUMO:Ubc9 complex that fuels Pli1 activity and, that Pli1 is degraded by STUbL activity in cells lacking Nup132 (human NUP133; [12, 29]). In agreement with these analyses, telomeres in SUMO^D81R^ and *nup132Δ* cells are longer than wild-type (Fig 6B). In contrast, the telomeres of *tetO7-SUMO*, *slx8-29* and *ufd1-1* cells are essentially wild-type in length (Fig 6B). Therefore, telomere length regulation through Pli1-dependent SUMOylation of Tpz1 [19, 20] is retained in *tetO7-SUMO* cells, as well as those with STUbL pathway dysfunction.

Interestingly however, telomeres in SUMO chain reducing SUMO^K14,30R^ cells are clearly elongated when compared to those in wild-type cells (Fig 6B). This unexpected finding either reveals a new role for SUMO chain-modified Tpz1 in telomere length maintenance, or indicates that SUMO lysine residues 14 and 30 are subject to other forms of regulatory posttranslational modification that impact telomere length.

### SUMOylation homeostasis at *taz1Δ* telomeres

**Fig 7:**
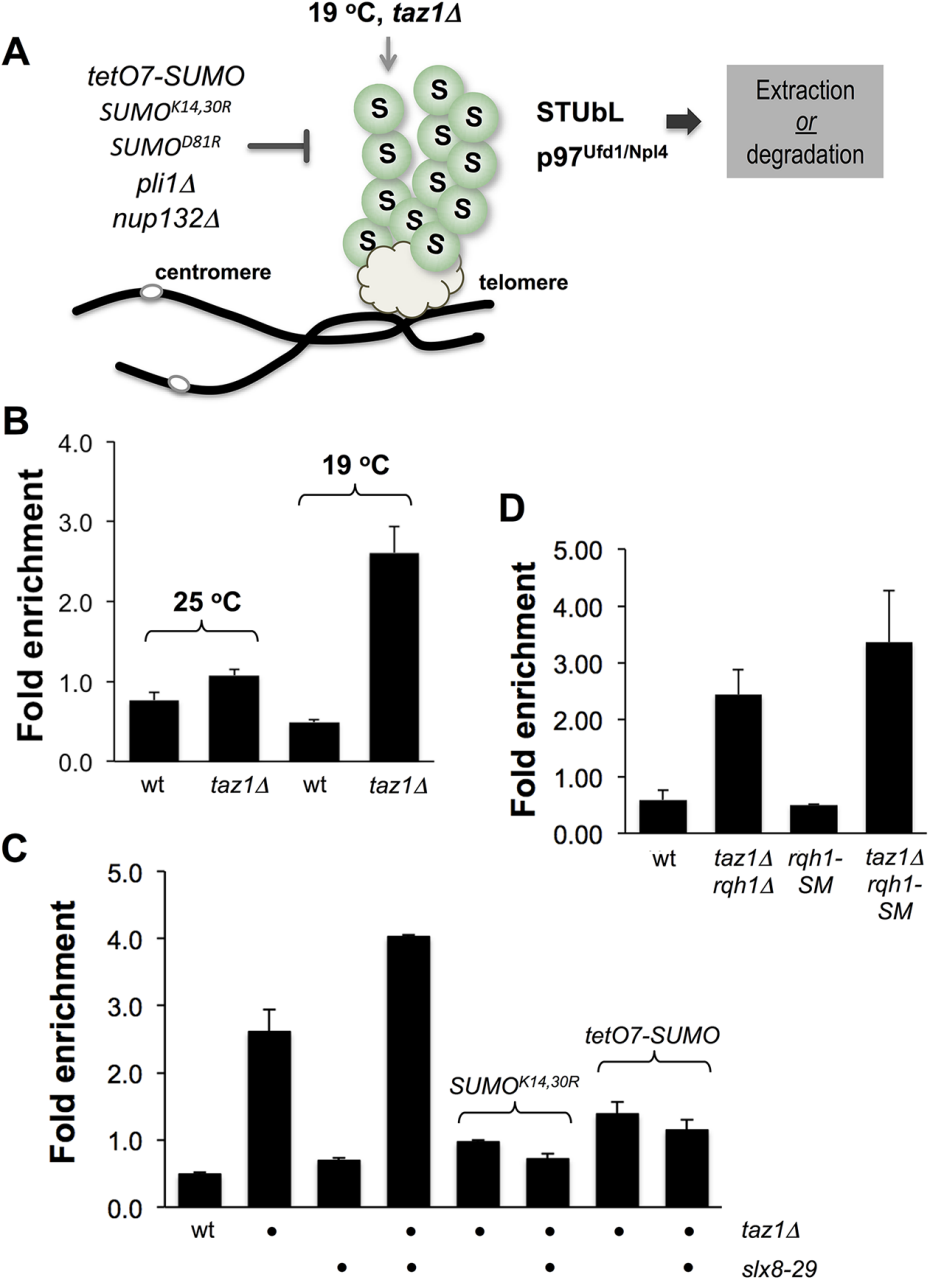
SUMO chain accumulation at *taz1Δ* telomeres. *A*, Our genetic analysis supports a model wherein Pli1-dependent SUMO chains persistently hyper-accumulate at telomeres in *taz1Δ* cells. Reducing SUMO chain formation by mutating Pli1, Nup132, or SUMO itself curbs SUMO chain accumulation at telomeres. In turn, this prevents the engagement of STUbL and Cdc48(p97) activities that drive telomere entanglement. *B*, *C*, *D*. ChIP analysis of SUMO at telomere of the indicated genotypes. Telomeric association of SUMO was measured by quantitative real-time PCR, and normalized against background (see Materials and Methods for details on calculation). Plots show mean values ± standard error for two or more independent experiments. Cells in *C* & *D* were grown at 19°C.

If SUMO chains promote STUbL activity at *taz1Δ* telomeres (e.g. Fig 7A), then elevated SUMO conjugates should be detectable at telomeres using anti-SUMO chromatin immunoprecipitation and quantitative PCR (ChIP-qPCR). Indeed, ChIP-qPCR revealed a strong enrichment of SUMO conjugates at *taz1Δ* versus wild-type telomeres, particularly in cells grown at 19°C (Fig 7B). These telomeric SUMO conjugates are predominantly chains, as they are reduced to near wild-type levels in both *taz1Δ tetO7-SUMO* and *taz1Δ SUMO*^*K14*,*30R*^ double mutant cells (Fig 7C).

Consistent with the known SUMO chain-directed activity of STUbL [7, 29, 38, 68], telomeric SUMO chains were further elevated in *taz1Δ slx8-29* double mutant over *taz1Δ* cells (Fig 7C). Therefore, SUMO chains generated on taz1*Δ* telomeres engage STUbL activity.

A SUMO conjugated form of RecQ helicase Rqh1 was shown to promote telomeric entanglement in *taz1Δ* cells [46]. Therefore, we considered the possibility that SUMOylated Rqh1 contributes to the elevated SUMO signal detected at *taz1Δ* telomeres (Fig 7B and 7C). However, neither *rqh1Δ* nor the Rqh1 SUMOylation deficient mutant Rqh1-SM [46] had a detectable affect on telomeric hyper-SUMOylation in *taz1Δ* cells (Fig 7D). Moreover, because Rqh1-SM rescues *taz1Δ* cold sensitivity [46], hyper-SUMOylation is not a consequence of Rqh1-dependent *taz1Δ* telomere entanglement (Fig 7D).

Together, our data indicate that the dysfunctional telomeres in *taz1Δ* cells are subjected to increased SUMO chain conjugation and subsequent STUbL activity. In turn, this likely promotes telomeric entanglements through downstream DNA repair factors such as Rqh1 [46].

## Discussion

The highly conserved STUbL family has critical roles in the DNA damage response (DDR) and genome stability [38, 48, 69]. Importantly, STUbL could support the DDR in two quite distinct ways (i) STUbL may act in a sequential DNA repair cascade to remove specific poly-SUMO conjugates in a "programmed manner" [48], or (ii) As the DDR promotes localized "group SUMOylation" of proteins at DNA lesions [70], we hypothesized that STUbL may also (or instead) antagonize local SUMO chain formation. If left unchecked by STUbL, these SUMO chains could inhibit downstream DDR events.

Together, our analysis supports inhibition of localized SUMO chain formation as a major role for STUbL in fission yeast. That is, if "programmed" SUMO chain and STUbL-dependent removal of DDR factors were critical for normal DNA repair, then blocking SUMO chain formation would cause DNA repair defects analogous to those of STUbL mutants. This is clearly not the case (see Fig 2 & [12, 30, 36, 71]). Moreover, inhibiting SUMO chain formation strongly reduces the need for STUbL in DNA repair, genome stability and cell growth (Fig 4 & [12, 30, 36, 71]). Therefore, although mono-SUMOylation plays critical roles in genome stability and the DDR, SUMO chain formation in STUbL mutant cells has a predominantly negative impact.

STUbL could antagonize SUMO chains in a number of ways including (i) its ability to "cap" SUMO chains by ubiquitinating the amino-terminal lysine residues that are acceptors for SUMO chain growth [35] (ii) its ability together with Cdc48/p97 to promote the local degradation/extraction of the SUMO ligases Pli1 and SIZ1 [29, 72] or (iii) its ability to degrade/extract other SUMOylated proteins that are "licensed" for SUMOylation e.g. MDC1 at DNA lesions, and therefore constantly nucleate the SUMOylation machinery [48].

**Fig 8:**
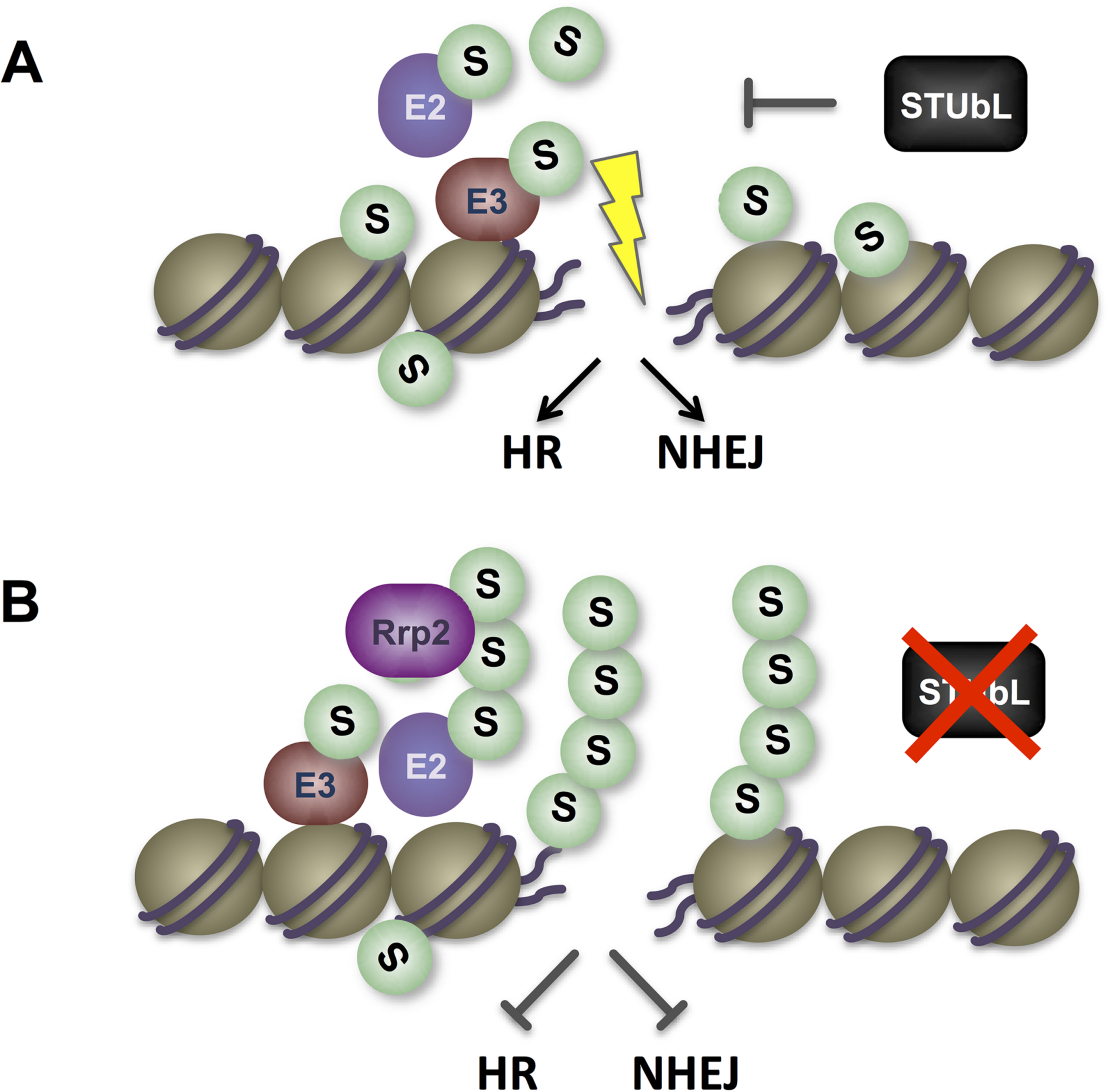
Model integrating known STUbL functions with data herein. *A*, STUbL and Cdc48(p97) activities are known to act at DNA lesions to make them permissive for downstream DDR events [48]. However, the nature of the DDR-inhibitory signal(s) upon STUbL and Cdc48(p97) inactivation remains unclear. We propose that SUMO chains are DDR-inhibitory, and their formation by the locally hyperactive SUMO conjugation pathway must be actively antagonized by STUbL and Cdc48(p97). STUbL could antagonize SUMO chains by (i) ubiquitinating SUMO's amino-terminal lysine residues and "capping" SUMO chain growth [35] (ii) driving the local degradation/extraction (via Cdc48/p97) of the major SUMO ligases Pli1 and SIZ1 [29, 72] or (iii) degrading/extracting other SUMOylated proteins that are "licensed" for SUMOylation e.g. MDC1 at DNA lesions, and therefore constantly nucleate the SUMOylation machinery [48]. *B*, in the absence of STUbL, SUMO chains accumulate and inhibit DNA repair including homologous recombination (HR) and non-homologous end joining (NHEJ) [48]. SUMO chains may exert intrinsic/steric DDR-inhibitory effects, but could also attract proteins such as the Swi2/Snf2 DNA translocase Rrp2 (and orthologs) that has known DNA repair modulatory functions. This model is consistent with the broadly compromised DDR in STUbL mutant cells, and suppression of this phenotype by reducing SUMO chain formation.

SUMO chains in STUbL mutant cells clearly have a negative effect on genotoxin resistance, but how they exert this effect was undefined. Intriguingly, we identified the Swi2/Snf2 translocase Rrp2 in a SUMO chain-binding proteomic screen [36], and further analysis indicates that it contributes to SUMO chain toxicity in STUbL mutant cells. Based on the known functions of Rrp2 (ScUls1) [55, 56, 60, 61] and our analysis of SUMOylation levels upon its deletion or overexpression, Rrp2 likely antagonizes STUbL activity at SUMO chains. In the future, it will be interesting to determine if Rrp2 also contributes to DNA repair inhibition by SUMO chains in *slx8-29* cells. In this regard, both Rrp2 and Uls1 have been shown to regulate DNA repair [55, 60]. For example, Uls1 inhibits the non-homologous end joining of telomeres in the presence of SUMO chains [60]. Furthermore, Rrp2 and Uls1 can inhibit Rad51-dependent homologous recombination [55, 61]. Therefore, Rrp2 and its orthologs in other species could contribute to the DDR inhibition observed upon STUbL inactivation (Fig 8).

So far, the analysis of SUMO chain function has depended on the use of SUMO mutants that reduce chain extension e.g. SUMO^K14,30R^ [12, 28, 50]. However, these mutants are refractory not only to modification by SUMO itself, but also by other posttranslational modifications such as ubiquitination. Indeed, STUbL ubiquitinates the lysine residues used for SUMO chain formation in budding yeast, thereby "capping" growth of SUMO chains [35].

Here, we show that limited expression of wild-type SUMO (*tetO7-SUMO*) provides robust suppression of the phenotypes caused by STUbL and Ulp2 mutants (Fig 2 & S1 Fig). Importantly, growth, genotoxin resistance and telomere length appear normal in *tetO7-SUMO* cells, demonstrating that key SUMOylation events required for genome stability are intact (Figs 2 & 6). For example, Tpz1 SUMOylation is lost in *pli1Δ* cells causing telomere elongation [19, 20], whereas *tetO7-SUMO* telomeres are wild-type in length. These striking results reinforce the model in which STUbL antagonizes local SUMO pathway activity at DNA lesions to prevent toxic SUMO chain buildup (Fig 8).

Surprisingly, unlike *tetO7-SUMO*, the SUMO chain blocking mutant SUMO^K14,30R^ causes telomere elongation. Therefore, although both *tetO7-SUMO* and SUMO^K14,30R^ largely bypass the need for STUbL or Ulp2 activity, the mutations in SUMO^K14,30R^ additionally impact Pli1 and Tpz1-dependent telomere length regulation [19, 20]. How SUMO^K14,30R^ impacts telomere length is unknown, but may be through defects in its ability to form short SUMO chains or to be ubiquitinated. This supports the utility of *tetO7-SUMO* in the analysis of SUMO pathway homeostasis, and in corroborating phenotypes ascribed to SUMO chains using SUMO^K14,30R^ and analogous mutants.

Analysis of *taz1Δ* telomere phenotypes provides new insight into dysfunctional telomere management by the SUMO pathway, and unexpected deleterious effects of STUbL activity. Taz1^TRF1/TRF2^-depleted telomeres become hyper-SUMOylated at low growth temperature (Fig 7). These telomeric SUMO conjugates contain SUMO chains, as they are strongly reduced in both *tetO7-SUMO* and SUMO^K14,30R^ backgrounds. Strikingly, the genome instability and cell death caused by unscheduled repair of *taz1Δ* telomeres is suppressed by mutations in the SUMO pathway that compromise SUMO chain-dependent STUbL activity. Our data indicate that Pli1-dependent SUMO chain formation engages STUbL and Cdc48 (p97) at *taz1Δ* telomeres (Fig 8). In turn, this makes *taz1Δ* telomeres permissive for pathological processing by Rqh1 and other DNA repair factors [46, 47].

STUbL-dependent processing of structurally distinct DNA lesions in budding yeast has recently been characterized [73–75]. A common theme is emerging: SUMO accumulates at persistent lesions such as irreparable DNA double strand breaks, eroded telomeres in cells lacking telomerase, or triplet repeat tracts that are difficult to replicate [73–75]. These heavily SUMOylated lesions are then processed by STUbL activity at the nuclear pore, promoting alternative repair pathways. Perhaps most relevant to our study is the STUbL-mediated processing of eroded telomeres in telomerase negative budding yeast, which promotes the emergence of so called Type II survivors [73]. Type II survivors maintain telomere terminal tracts in manner dependent on a number of DNA repair factors, including the RecQ helicase Sgs1. In this regard, it is noteworthy that the fission yeast Sgs1 homolog, Rqh1, promotes *taz1Δ* telomere entanglement [46]. Therefore, it is tempting to speculate that STUbL processing of short eroded telomeres (telomerase delete), or those that are highly elongated and difficult to replicate (*taz1Δ*), allows access to similar DNA repair machinery, including RecQ helicases. In the case of short eroded telomeres, this RecQ-dependent processing is beneficial to the cell and improves fitness [73], whereas it entangles the elongated telomeres of *taz1Δ* cells, leading to genome instability.

Analysis of cytotoxic Top1-DNA adduct (Top1cc) processing provides additional evidence for SUMO chain-dependent inhibition of DNA repair in STUbL mutant cells (Fig 2D; [22, 36]). We showed that STUbL and Cdc48(p97) cooperate in Top1cc repair in cells that lack the Top1cc reversing enzyme Tdp1 [36]. Combining STUbL and Tdp1 mutations causes Top1-dependent genome instability and hypersensitivity to camptothecin [36, 76]. Importantly, the sickness of *slx8-29 tdp1Δ* cells is strongly suppressed by *tetO7-SUMO*, and albeit less efficiently, by SUMO^K14,30R^ (Fig 2D; [22, 36]). Therefore, the accumulation of SUMO chains in *slx8-29 tdp1Δ* cells inhibits Top1cc repair by the alternative mechanism i.e. Rad16-Swi10^XPF-ERCC1^ cleavage [22, 36]. Deleting the SUMO chain-binding DNA translocase Rrp2 also partially suppresses the *slx8-29 tdp1Δ* phenotype (Fig 3), revealing a candidate mediator of repair inhibition in this context.

In the absence of exogenous stress, STUbL may also maintain the functionality of chromatin loci that are normally heavily SUMOylated e.g. heterochromatic centromeres [14, 18, 77]. Indeed, in a recent proteomic analysis of STUbL and Cdc48 substrates in fission yeast, proteins located at centromeres and telomeres were over represented [40]. Moreover, deleting the major SUMO E3 ligase for these loci, Pli1, bypasses the essential role(s) of STUbL in fission yeast (this study & [30]).

Overall, integrating our current data with the known DDR-inhibitory effects of STUbL and Cdc48/p97 dysfunction (e.g. [42–45, 48, 49]), unchecked SUMO chains at DNA lesions appear to antagonize downstream repair processes (Fig 8). In this setting, the Rrp2 DNA translocase emerges as an excellent candidate mediator of SUMO chain toxicity. We also reveal that STUbL activity can destabilize the genome, depending on the nature of the initial DNA lesion.

Given the highly conserved nature of STUbL and its cofactors, antagonizing localized SUMO chain accumulation is likely to be a conserved role of STUbL across species.

## Materials and methods

### Yeast strains, plasmids, and general methods

Standard media and growth conditions for *S*. *pombe* were used as described previously [78]. All strains (Table 1) are of genotype *ura4-D18 leu1-32* unless otherwise stated. For Spot Assays, cells were grown at 25°C to logarithmic phase (optical density at 600 nm [OD_600_] of 0.6 to 0.8), spotted in 5-fold dilutions from a starting OD_600_ of 0.5 on plates supplemented with the relevant drug. The plates were incubated at 19 to 35°C for 3 to 6 days.

**Table 1:** A list of yeast strains used in this study.

Strain	Genotype	Source
NBY780	*h*^*+*^	
NBY1457	*SUMO*::*ura4+*	
NBY1493	*pli1*::*kanMx6*	
NBY1671	*pli1*::*hphMx6*	
NBY2079	*tdp1*::*ura4*^*+*^	
NBY2429	*tdp1*::*ura4*^*+*^ *slx8-29*:*kanMx6*	
NBY2471	*slx8-29*:*kanMx6*	
NBY2475	*slx8-29*:*kanMx6 pli1*::*hphMx6*	
NBY2499	*slx8-29*:*hphMx6*	
NBY2756	*slx8-29*:*kanMx6 pREP2*:*ura4*^*+*^ *integrated at ars1*	
NBY2922	*ura4*^*+*^ *leu1*^*+*^	
NBY3172	*ulp2*::*hphMx6*	
NBY3401	*SUMO*^*D81R*^	
NBY3232	*SUMO*^*K14*,*K30R*^	
NBY3272	*SUMO*^*K14*,*K30R*^ *slx8-29*:*kanMx6*	
NBY4062	*ufd1-1*:*kanMx6 pREP2*:*ura4*^*+*^ *integrated at ars1*	
NBY4928	*taz1*::*ura4*^*+*^	
NBY4929	*taz1*::*ura4*^*+*^ *slx8-29*:*kanMx6*	
NBY4930	*taz1*::*ura4*^*+*^ *ufd1-1*:*kanMx6*	
NBY4975	*taz1*::*ura4*^*+*^ *slx8-29*:*kanMx6* pREP41*-mCherry*:*LEU2*	
NBY4976	*taz1*::*ura4*^*+*^ *slx8-29*:*kanMx6* pREP41*-GFP-slx8*:*LEU2*	
NBY4977	*taz1*::*ura4*^*+*^ *slx8-29*:*kanMx6* pREP41*-GFP-RNF4*:*LEU2*	
NBY4978	*taz1*::*ura4*^*+*^ *ufd1-1*:*kanMx6* pREP41*-mCherry*:*LEU2*	
NBY4979	*taz1*::*ura4*^*+*^ *ufd1-1*:*kanMx6* pREP41*-mCherry-ufd1*:*LEU2*	
NBY4980	*taz1*::*ura4*^*+*^ *slx8-29*:*kanMx6 SUMO*^*K14*,*K30R*^	
NBY5074	*tetO*_*7*_*-TATA*_*CYC1*_*SUMO*:*kanMx*	
NBY5105	*tetO*_*7*_*-TATA*_*CYC1*_*SUMO*:*kanMx slx8-29*:*hphMx6*	
NBY5114	*tetO*_*7*_*-TATA*_*CYC1*_*SUMO*:*kanMx ulp2*::*hphMx6*	
NBY5116	*tetO*_*7*_*-TATA*_*CYC1*_*SUMO*:*kanMx taz1*::*ura4*^*+*^	
NBY5427	*nup132*::*ura4*^*+*^	
NBY6041	*tetO*_*7*_*-TATA*_*CYC1*_*SUMO*:*kanMx pREP2*:*ura4*^*+*^ *integrated at ars1*	
NBY5557	*tetO*_*7*_*-TATA*_*CYC1*_*SUMO*:*kanMx tdp1*::*ura4*^*+*^ *slx8-29*:*hphMx6*	
NBY5786	*LacI-GFP*:*arg3*^*+*^ *LacO-Cen1*:*lys1*^*+*^ pJK148-P_*nmt8*_*-LacI-mCherry-SUMO*_*GG*_ *integrated at leu1*^*+*^	
NBY5763	*LacI-GFP*:*arg3*^*+*^ *LacO-cen1*:*lys1*^*+*^ *slx8-29*:*kanMx6* pJK148-P_*nmt8*_*-LacI-mCherry-SUMO integrated at leu1*^*+*^	
NBY5827	*taz1*::*ura4*^*+*^ *SUMO*^*K14*,*K30R*^	
NBY5841	*tdp1*::*ura4*^*+*^ *SUMO*^*K14*,*K30R*^ *slx8-29*:*kanMx6*	
NBY5850	*LacI-GFP*:*arg3*^*+*^ *LacO-cen1*:*lys1*^*+*^ *slx8-29*:*kanMx6* pJK148-P_*nmt8*_*-LacI-mCherry- SUMO*^*K14*,*K30R*^ *integrated at leu1*^*+*^	
NBY5925	*taz1*::*ura4*^*+*^ *pli1*::*kanMx6*	
NBY5934	*taz1*::*ura4*^*+*^ *nup132*::*hphMx6*	
NBY5935	*taz1*::*ura4*^*+*^ *SUMO*^*D81R*^	
NBY6060	*taz1*::*ura4*^*+*^ *slx8-29*:*hphMx6 tetO*_*7*_*-TATA*_*CYC1*_*SUMO*:*kanMx*	
NBY6069	*rrp2*::*kanMx6*	
NBY6116	*rrp2*::*kanMx6 slx8-29*:*hphMx6*	
NBY6121	*rqh1-SM23*	[46]
NBY6122	*taz1*::*ura4*^*+*^ *rqh1-SM23*	[46]
NBY6133	*rrp2*::*kanMx6 tdp1*::*ura4*^*+*^	
NBY6134	*rrp2*::*kanMx6 tdp1*::*ura4*^*+*^ *slx8-29*:*hphMx6*	
NBY6135	*taz1*::*ura4*^*+*^ pREP41:*LEU2*	
NBY6136	*taz1*::*ura4*^*+*^ pREP41-Pli1:*LEU2*	
NBY6137	*taz1*::*ura4*^*+*^ *pli1*::*kanMx6* pREP41:*LEU2*	
NBY6138	*taz1*::*ura4*^*+*^ *pli1*::*kanMx6* pREP41-Pli1:*LEU2*	
NBY6140	*pli1*::*kanMx6* pREP41-mCherry-FLAG-Rrp2:*LEU2*	
NBY6141	*SUMO*^*K14*,*K30R*^ pREP41-mCherry-FLAG-Rrp2:*LEU2*	
NBY6142	*tetO*_*7*_*-TATA*_*CYC1*_*SUMO*:*kanMx* pREP41-mCherry-FLAG-Rrp2:*LEU2*	
NBY6145	*rqh1*::*kanMx6*	
NBY6153	pREP41-mCherry-FLAG-Rrp2:*LEU2*	

The chimera LacI-mCherry-SUMO was cloned by serial insertions of LacI and mCherry-SUMO into a pREP81 vector at the NdeI, then SalI and BamHI sites, respectively. The primers used are listed in Table 2. The *E*. *coli* LacI with the last five amino acid truncated to disable tetramerization [79] was amplified using Omn406 and Omn411 primers from the *E*. *coli* genome. LacI was cloned in frame with mCherry-SUMO_GG_ amplified using Omn083 and Omn408 from pREP41-mCherry-SUMO (pMN087). The sequence containing the P_*nmt8*_ promoter and LacI-mCherry-SUMO_GG_ was amplified using Omn423 and Omn424 primers, and transferred, by Gibson Assembly [80], to pJK148 that has been linearized with PstI and SacI, to generate pNB075. To mutagenize SUMO from wild-type to SUMO^K14,30R^, site-directed mutagenesis was performed using the JP243/ JP244 (K14R), oNB112/oNB113 (K30R) primers to make pJK148-P_*nmt8*_-LacI-mCherry-SUMO^K14,30R^ (pNB077).

**Table 2:** A list of the oligonucleotides used in this study.

Primer	Sequence
JP243	5’-CATTTCTGATGCTGACAGAAGTGCTATCACTCCTACC-3'
JP244	5’- GGTAGGAGTGATAGCACTTCTGTCAGCATCAGAAATG-3'
Omn083	5’- GACTAGGATCCCTAACCACCTAACTGTTCTAAGACAGC-3'
Omn228	5’- CGCCGAACGTGAAATTGTTCGTGA-3'
Omn229	5’-TCAAGGGAGGAAGATTGAGCAGCA-3'
Omn406	5’-AGATTCATATGAAACCAGTAACGTTATACGATG-3'
Omn408	5’- GATTATGTCGACTGTGAGCAAGGGCGAGGAG-3'
Omn411	5’- GACTACATATGTCGGGAAACCTGTCGTGCC-3'
Omn423	5’- TTGATATCGAATTCCTGCAGGTCGATCGACTCTAGAGGATCA-3'
Omn424	5’-GGGAACAAAAGCTGGAGCTCGTGTCAGATAAGTCACTATGTCC-3’
oNB112	5’- ACACTTCGCAACAAGATGTCAGACCATCCACAGAGCATATCAA-3'
oNB113	5’- TTGATATGCTCTGTGGATGGTCTGACATCTTGTTGCGAAGTGT-3'

The pNB075 or pNB077 plasmid was linearized by digesting with NruI and integrated at the *leu1* locus by homologous recombination.

### Microscopy

For live imaging, cells were grown in filter-sterilized EMM medium supplemented with leucine, uracil, arginine, and histidine (LUAH) to logarithmic phase. Bright-field and fluorescence images of live cells were acquired using a Nikon Eclipse microscope with a 100x Plan Apochromat DIC H oil immersion objective and a Photometrics Quantix charge-coupled device camera. Images were analyzed with NIH ImageJ software.

### Western blotting

TCA precipitation of total proteins and Western blotting were carried out as previously described [29]. The membrane was blocked in 1% *w/v* non-fat milk in phosphate buffer saline solution with 0.1% *v/v* Tween-20, probed with antibodies against α-tubulin (Sigma) and *S*. *pombe* SUMO [12], followed by HRP- or IRDye-conjugated secondary antibodies, and detected either using an ECL Dura system (Pierce) on film; or scanning on an ODYSSEY scanner (Li-Cor).

### Southern blot analysis

Fission yeast genomic DNA was digested with EcoRI, resolved on a 1% agarose gel, and then transferred onto a Hybond-XL membrane (GE Healthcare) using a TurboBlotter (Whatman). The membrane was UV autocrosslinked at 120 mJ/cm^2^ (Stratalinker 1800). The 0.3-kb ApaI-EcoRI fragment of pTELO [81] was used as a template to generate a probe using the Takara Random Primer DNA Labeling Kit (Takara).

### ChIP assays and quantitative PCR

Logarithmic phase cells (25 OD_600_ units) were fixed in 1% (w/v) formaldehyde for 25 min at room temperature. The reaction was stopped by adding 2.5 M glycine to a final concentration of 125 mM and incubate for 5 min at room temperature while shaking. Cells were washed in ice-cold Tris-buffered saline (TBS) solution. Cell pellet was resuspended in 0.4 mL of FA lysis buffer (50 mM HEPES, pH 7.6, 150 mM NaCl, 1 mM EDTA, 0.1% sodium deoxycholate, 1% Triton X-100, 0.1% SDS), supplemented with 1 mM PMSF, 20 mM N-ethylmaleimide, and Complete protease inhibitor tablet EDTA-free (Roche), and lysed by beating with silica–zirconia beads four times at 5.0 m/s for 20 s in a FastPrep-24 (MP Biochemicals). After clarification by centrifugation for 10 min at 16,000 x g in a microfuge at 4°C, pellet was re-suspended in 0.3 mL of the supplemented FA lysis buffer. After sonication for 30 s (30 s on, 30 s off) in 16 cycles using a Bioruptor Pico (Diagenode), the average size of sheared chromatin was less than ~300 bp, and subsequently cleared of insoluble cell debris by centrifugation at 16,000 x g for 10 min. After adding FA lysis buffer to bring the total volume to 1 mL, 10 μL of the clarified extract was saved as an input control. Immunoprecipitation was performed at 4°C for 2 h with protein G magnetic Dynabeads (Thermofisher) that had been coupled with α-SUMO antibody (1 μg per sample). Reverse crosslinking was performed as described [81].

Recovered DNA from input or ChIP was used as template for SYBR Green-based real-time PCR (Bio-Rad). Fold enrichment values were calculated based on *Δ*Ct between ChIP and input using the jk380/jk381 primer pairs of subtelomere [81] and an *act1* (actin) gene fragment as background control (Omn228, Omn229 primers, Table 2). The values were expressed as ChIP/input (subtelomere) normalized with ChIP/input (act1^+^) as described [82].

## Supporting information

S1 Fig *A*, five fold serial dilutions of the indicated strains were spotted onto YES plates, with or without the indicated genotoxic challenge, and grown at indicated temperature. *B*, anti-SUMO and tubulin Western blots of total proteins from the indicated strains grown at 25°C to OD_600_ of 0.2, then at 35°C for six hours before harvesting.
(TIFF)
