# Supplementary figures and images for: SUMO-targeted ubiquitin ligase activity can either suppress or promote genome instability, depending on the nature of the DNA lesion
Source: PLoS Genet. 2017 May 5;13(5):e1006776. doi: 10.1371/journal.pgen.1006776 (PMC5438191; doi:10.1371/journal.pgen.1006776)

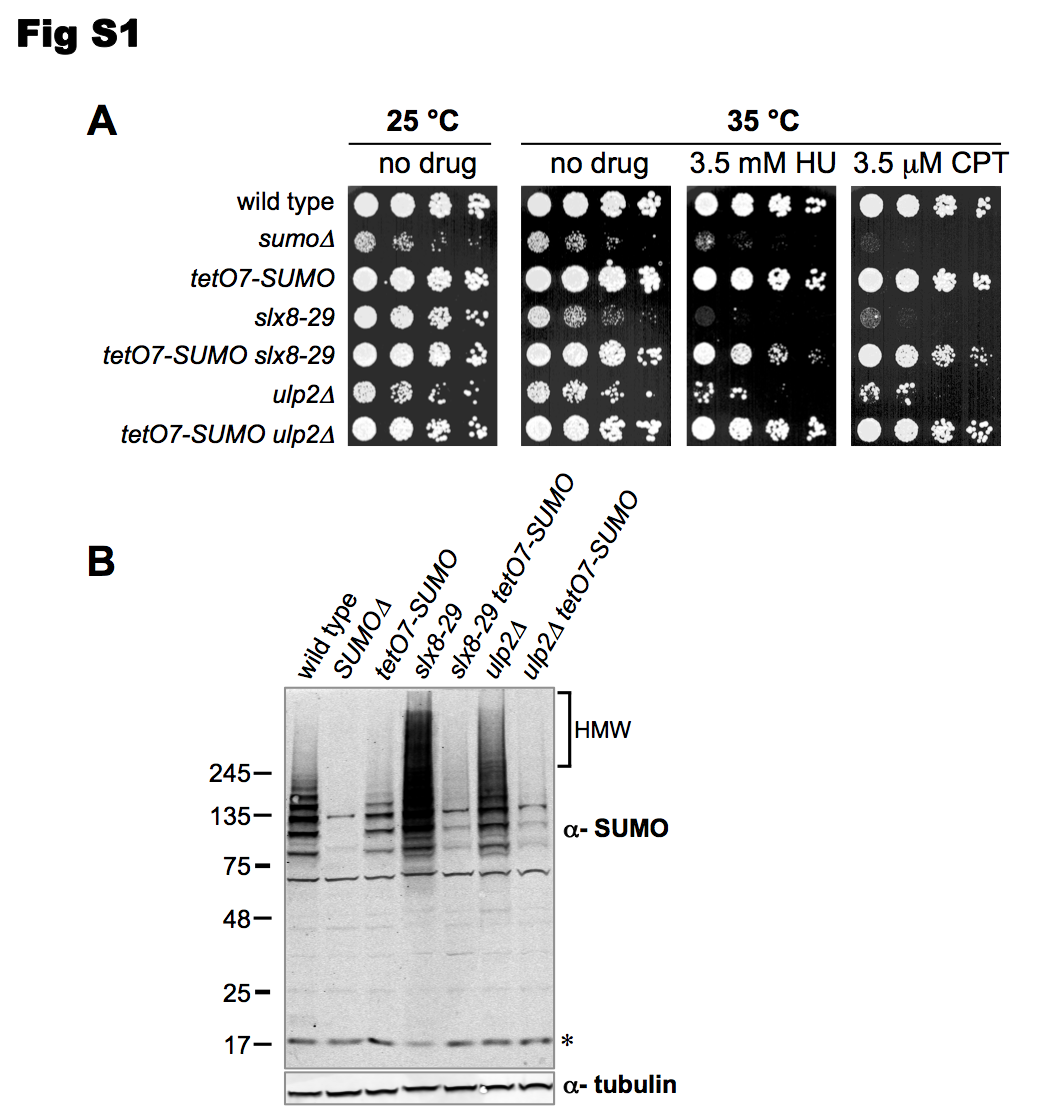

Supplement: S1 Fig — A, five fold serial dilutions of the indicated strains were spotted onto YES plates, with or without the indicated genotoxic challenge, and grown at indicated temperature. B, anti-SUMO and tubulin Western blots of total proteins from the indicated strains grown at 25°C to OD600 of 0.2, then at 35°C for six hours before harvesting. (TIFF) [file pgen.1006776.s001.tiff]
